# A survey on social support during standardized training for pediatric residents in Southwest China: a preliminary study

**DOI:** 10.3389/fmed.2026.1696233

**Published:** 2026-02-04

**Authors:** Hongdong Li, Jie Liu, Han Wang, Liping Tan

**Affiliations:** 1 Department of Emergency, Children's Hospital of Chongqing Medical University, Chongqing, China; 2 National Clinical Research Center for Child Health and Disorders, Chongqing, China; 3 Ministry of Education Key Laboratory of Child Development and Disorders, Chongqing, China; 4 Chongqing Key Laboratory of Child Rare Diseases in Infection and Immunity, Chongqing, China

**Keywords:** pediatric, resident, social support, standardized training, survey

## Abstract

**Background:**

There is little data regarding social support for pediatric residents in standardized training. The aim of this study was to understand the current status and factors influencing social support during standardized training for pediatric residents in Southwest China.

**Methods:**

An electronic survey was administered to pediatric residents from all three professional years at the Children's Hospital of Chongqing Medical University in Chongqing, Southwest China. Pediatric residents anonymously self-provided their demographic information along with social support ratings using the Social Support Rating Scale (SSRS), and multiple logistic regression analysis was performed to evaluate the factors influencing overall social support.

**Results:**

A total of 223 pediatric residents completed the survey and provided valid questionnaires. Overall, 76.23% (170/223) of the participants were satisfied with the level of social support they received. Female pediatric residents demonstrated significantly greater utilization of social support and stronger overall support levels (*P* < 0.05). Additionally, compared with those who did not have a medical license, pediatric residents who had obtained a physician's medical license reported significantly higher social support levels (*P* < 0.05). The factors influencing social support were gender, employment and a physician's medical license.

**Conclusions:**

The level of social support for pediatric residents remains suboptimal, with particular attention required for male pediatric residents and those who have not yet obtained their practicing qualifications. It is necessary to formulate and execute better standardized training policies for pediatric residents, increase social support and improve training quality.

## Introduction

In recent years, the shortage of doctors has become increasingly severe worldwide, particularly in low- and middle-income countries, with pediatricians being especially scarce (1). The American Academy of Pediatrics has also warned that the United States could face a shortage of up to 55,000 pediatricians over the next decade (2). Research conducted between 2015 and 2016 revealed that there were only four pediatricians per 10,000 children in China, far from the target number (3). However, China's pediatrician attrition rate has reached a staggering 12.6% per year, with this phenomenon being particularly prevalent among young resident physicians undergoing standardized training (3).

China's standardized residency training program was officially launched in 2014 and has been continuously improved (4). This program plays a vital role in helping resident physicians improve their clinical skills and accumulate practical experience, serving as a crucial bridge for their lifelong learning and professional development (5). The same applies to pediatricians—whether they hold bachelor's, master's, or doctoral degrees, they must complete a three-year standardized training program.

With rapid socioeconomic development of China, people's living standards have reached unprecedented levels, leading to heightened awareness of personal health and increased healthcare-seeking behaviors. As front-line clinicians, resident physicians undergoing standardized training frequently interact with patients and shoulder heavy clinical workloads. However, due to their relative lack of clinical experience compared with seasoned physicians, insufficient proficiency in practical skills, and limited patient communication skills, these residents find themselves in a transitional predicament (6). In the face of these professional demands, this group is particularly vulnerable to high stress levels, insomnia, emotional instability, and subsequent occupational burnout or depression (7). Such psychological distress may adversely affect both the trainees themselves and their patients (8, 9).

Social support is a protective factor of psychological issues such as occupational burnout, and increasing social support can reduce the occurrence of these issues (10). Social support refers to the tangible or perceived material, emotional, and psychological assistance an individual receives from their external environment (11). It can be categorized as subjective support or objective support. Subjective support pertains to the emotional backing an individual perceives, such as understanding and respect from family and friends. Objective support involves concrete material assistance, such as financial aid provided by enterprises, institutions, or social organizations (12). Social support theory was developed to encourage society to extend all possible help to disadvantaged groups, enabling them to maintain normal daily functioning and adopt a more proactive approach to life's challenges (12). Social support can mitigate the adverse effects of stress on mental health, with lower support levels correlating with greater psychological distress (13, 14). Studies have found that individuals with weaker social support are five times more likely to exhibit symptoms of anxiety and depression than are those with stronger support networks (11).

Research on social support for pediatricians remains scarce. Song et al. (7) found that psychological resilience and burnout play a chain-mediating role in the association between social support and negative emotions. However, their epidemiological findings on social support surveys among pediatricians remain unmeasured. To fill this gap in measurement, it is imperative to conduct a social support survey of pediatric resident physicians undergoing standardized training in China to obtain scientific evidence for reforming pediatric residency education, developing talent retention strategies, and optimizing healthcare policies.

## Materials and methods

### Settings and participants

This cross-sectional survey was conducted at the Children's Hospital of Chongqing Medical University in Chongqing, Southwest China, between March 2023 and April 2023. A total of 223 pediatric residents, including those at the bachelor's, master's, and doctoral levels, participated in this survey. This study was reviewed and approved by the Institutional Review Board of the Children's Hospital of Chongqing Medical University (No. 2023-IRB-66). All participants provided written informed consent in compliance with the Declaration of Helsinki.

### Questionnaire

Questionnaires were sent to all pediatric residents through Questionnaire Star, and the purpose of the questionnaire was explained before the questions were given. The questionnaire required approximately 30 min to complete. The participants were instructed to complete the questionnaire independently. After the pediatric residents completed the survey, the data were transmitted to our system for further analysis.

The questionnaire was used to collect demographic information, including gender, age, educational level, year of residency training, annual income, permanent residence address, and qualification and specialty area in residency training.

The Social Support Rating Scale (SSRS), developed by the Chinese researcher Shuiyuan Xiao (15), comprises ten items across three distinct dimensions: objective social support (four items), subjective social support (four items), and utilization of social support (three items). Items 1–5 and 8–10 use a 4-point Likert scale (1 = “not at all” to 4 = “very much”). Items 6 and 7 are binary coded (0 = “no source,” 1 = “have a source” per available source). Higher total scores reflect greater perceived social support, categorized as follows: < 20 (less social support), 20–30 (general social support), and >30 (satisfactory social support). The scale reliability in this study was excellent (Cronbach's alpha = 0.94).

### Statistical analysis

Statistical analyses were performed using SPSS software version 26.0 (SPSS, Inc., Chicago, IL). Abnormally distributed data are described using the median and interquartile range (IQR: 25–75%), and the Mann–Whitney *U*-test was used to assess differences between two groups. Descriptive statistics included frequencies (%) for categorical variables, and the chi-square test and Fisher's exact test were used to assess the groups' differences as appropriate. Multiple logistic regression analysis was used to evaluate the factors influencing overall social support. *P* < 0.05 was considered to indicate statistical significance.

## Results

### General characteristics of pediatric residents

A total of 223 pediatric residents completed the questionnaire, including 149 (66.82%) women and 74 (33.18%) men, with an average age of 24 years (Table 1). Among these participants, 31 were married (13.90%), and 192 were single (86.10%). There were 22 (9.87%) participants with a bachelor's degree, 156 (69.96%) with a master's degree and 45 (20.17%) with a doctoral degree. Among the 223 residents, 73 (32.74%) were in their first year of training, 80 (35.87%) were in their second year, and 70 (31.39%) were in their third year. The proportions of rural-oriented pediatric residents and urban pediatric residents were 60.99% and 39.01%, respectively. A total of 152 (68.16%) pediatric residents had obtained a physician's medical license, while 71 (31.84%) pediatric residents did not. The percentage of residents satisfied with the overall social support they received during their residency training was 76.23% (170/223).

**Table 1:** Demographic characteristics of the respondents (*n* = 223).

**Demographic characteristic**	***n* (%)**
**Gender**	
Male	74 (33.18)
Female	149 (66.82)
**Age**	
≤ 25	122 (54.71)
25–30	64 (28.70)
>30	37 (16.59)
**Education**	
Bachelor's	22 (9.87)
Master's	156 (69.96)
Doctorate	45 (20.17)
**Level of training**	
First year	73 (32.74)
Second year	80 (35.87)
Third year	70 (31.39)
**Marital status**	
Married	31 (13.90)
Unmarried	192 (86.10)
**Residence**	
Rural	136 (60.99)
Urban	87 (39.01)
**Annual income (RMB)**	
< 50,000	165 (73.99)
50,000–100,000	39 (17.49)
>100,000	19 (8.52)
**Physician's medical license**	
Yes	152 (68.16)
No	71 (31.84)

### The status of social support among pediatric residents

The mean scores on the SSRS and its three dimensions according to demographic characteristics are displayed in Table 2. Compared with male pediatric residents (5.00; IQR = 3.00–7.00), female pediatric residents (8.00; IQR = 7.00–9.00) reported higher scores for utilization of support (*P* < 0.05), but no differences in subjective support, objective support or total scores were observed (*P* > 0.05). There were no significant differences in scores for education, level of training, or annual income among these pediatric residents. However, compared to other doctors who were not employed by the Children's Hospital of Chongqing Medical University, in-house doctors employed by the Children's Hospital of Chongqing Medical University had higher objective social support scores (8.00; IQR = 3.00–9.00 vs. 5.50; IQR = 3.00–8.00; *P* < 0.05). Moreover, pediatric residents who obtained a physician's medical license had higher scores for subjective social support than did those without a medical license (21.00; IQR = 17.00–26.00 vs. 19.00; IQR = 15.00–22.00; respectively, *P* < 0.05).

**Table 2:** The status of social support among pediatric residents.

**Variable**	**Objective social support**		**Subjective social support**		**Utilization of social support**		**Total score**	
	**Median (IQR)**	***P***	**Median (IQR)**	***P***	**Median (IQR)**	***P***	**Median (IQR)**	***P***
**Gender**								
Male	6.00 (3.50–8.00)	0.14	19.00 (17.00–22.50)	0.59	5.00 (3.00–7.00)	0.01	31.00 (27.50–37.00)	0.14
Female	7.00 (5.00–8.00)		20.00 (16.8–23.00)		8.00 (7.00–9.00)		34.00 (30.00–39.00)	
**Education**								
Bachelor's	8.50 (6.50–9.00)	0.21	24.00 (20.30–26.80)	0.52	7.50 (6.75–8.50)	0.14	41.00 (33.50–45.30)	0.39
Master's	7.00 (5.00–8.00)		19.50 (17.00–22.00)		8.00 (6.75–9.00)		34.00 (30.00–38.30)	
Doctorate	6.00 (4.00, 8.00)		19.00 (16.00, 24.00)		7.00 (6.00, 9.00)		33.00 (27.00, 38.50)	
**Level of training**								
First year	6.00 (4.00–8.00)	0.80	19.00 (15.50–23.00)	0.16	7.00 (6.00–9.00)	0.36	33.00 (26.00–38.50)	0.42
Second year	6.00 (4.00–9.00)		20.00 (18.00–24.00)		7.00 (6.00–9.00)		35.00 (30.00–39.00)	
Third year	6.50 (5.00–8.00)		19.00 (16.80–22.00)		8.00 (7.00–9.00)		32.50 (29.00–37.30)	
**Annual income (RMB)**								
< 50,000	7.00 (5.00–8.00)	0.33	19.00 (17.00–23.00)	0.70	7.00 (6.00–9.00)	0.22	34.00 (30.00–38.00)	0.46
50,000–100,000	5.00 (3.00–9.00)		19.00 (16.00–23.00)		7.00 (6.00–10.00)		33.00 (26.00–39.00)	
>100,000	9.00 (6.00–10.00)		26.00 (20.00–27.50)		8.00 (7.50–10.00)		46.00 (35.00–47.50)	
**Resident type**								
In-house doctor	8.00 (3.00–9.00)	0.05	24.00 (21.00–26.00)	0.26	9.00 (8.00–9.00)	0.11	41.00 (33.00–45.00)	0.36
Other	5.50 (3.00–8.00)		19.50 (16.00–26.00)		7.00 (6.00–8.00)		31.50 (26.80–39.00)	
**Physician's medical license**								
No	6.00 (4.00–8.00)	0.43	19.00 (15.00–22.00)	0.05	7.00 (6.00–9.00)	0.10	32.00 (28.00–38.80)	0.08
Yes	6.00 (5.00–8.00)		21.00 (17.00–26.00)		8.00 (6.00–9.00)		34.00 (30.00–39.00)	

IQR, interquartile range. In-house doctor refers to pediatric residents who have been employed at the Children's Hospital of Chongqing Medical University.

The degree of social support among pediatric residents was also studied. The results for pediatric residents who were female, who were in-house doctors and who obtained physicians' medical license were significantly different from those for the other participants (*P* < 0.05; Table 3). However, no significant differences in education level, training, or annual income were observed (*P* > 0.05).

**Table 3:** The degree of social support among pediatric residents (*n* = 223).

**Variable**	**Less social support *n* (%)**	**General social support *n* (%)**	**Satisfactory social support *n* (%)**	***P***
**Gender**				
Male	5 (2.24%)	21 (9.42%)	48 (21.52%)	0.02
Female	0 (0.00%)	27 (12.11%)	122 (54.71%)	
**Education**				
Bachelor's	0 (0.00%)	7 (3.14%)	15 (6.73%)	0.09
Master's	0 (0.00%)	33 (14.80%)	123 (55.16%)	
Doctorate	5 (2.24%)	22 (9.87%)	18 (8.07%)	
**Level of training**				
First year	5 (2.24%)	24 (10.76%)	44 (19.73%)	0.28
Second year	0 (0.00%)	24 (10.76%)	56 (25.11%)	
Third year	0 (0.00%)	25 (11.21%)	45 (20.18%)	
**Annual income (RMB)**				
< 50,000	3 (1.35%)	35 (15.70%)	127 (56.95%)	0.16
50,000–100,000	1 (0.45%)	15 (6.73%)	23 (10.31%)	
>100,000	0 (0.00%)	3 (1.35%)	16 (7.17%)	
**Resident type**				
In-house doctor	0 (0.00%)	5 (2.24%)	35 (15.70%)	0.03
Others	15 (6.73%)	38 (17.04%)	130 (58.30%)	
**Physician's medical license**				
No	5 (2.24%)	28 (12.56%)	38 (17.04%)	0.02
Yes	0 (0.00%)	34 (15.25%)	118 (52.91%)	

### The factors influencing social support among pediatric residents

Multiple logistic regression was applied to identify the factors influencing social support. Compared with male pediatric residents, female pediatric residents were three times more likely to report being satisfied with the level of social support (OR = 3.12; 95% CI: 1.75–5.23; *P* < 0.001; Table 4). Additionally, the probability of being satisfied with the level of social support was almost three times greater for in-house doctors than for residents who were not employed by the Children's Hospital of Chongqing Medical University (OR = 2.88; 95% CI: 1.76–4.05; *P* = 0.005). Furthermore, the odds of being satisfied with the level of social support were five times higher for pediatric residents who acquired a physician's medical license than for those who did not (OR = 5.35; 95% CI: 3.19–8.98; *P* = 0.001). However, variables such as education and level of training were not found to influence social support.

**Table 4:** Multiple logistic regression analysis of the factors influencing perceived social support.

**Variable**	**β**	**OR**	**95% CI**	***P***
**Gender**				
Female	1.138	3.12	1.75–5.23	< 0.001
Male				
**Education**				
Bachelor's	0.626	1.87	0.95–3.02	0.31
Master's	0.351	1.42	0.63–2.86	0.43
Doctorate				
**Level of training**				
First year	−0.031	0.97	0.88–1.75	0.75
Second year	0.425	1.53	0.73–3.19	0.25
Third year				
**Resident type**				
In-house doctor	1.058	2.88	1.76–4.05	0.005
Others				
**Physician's medical license**				
Yes	1.677	5.35	3.19–8.98	0.001
No				

OR, odds ratio; CI, confidence interval.

## Discussion

A growing body of evidence indicates that social support plays a beneficial role in psychological wellbeing and overall quality of life. Strengthening social support systems can thus contribute to improved mental health outcomes and enhanced life satisfaction among individuals (16, 17). In this study, we explored social support status among pediatric residents in Southwest China, with an emphasis on investigating the factors influencing perceived social support. The results demonstrated that the percentage of residents satisfied with the overall social support they received during their residency training was 76.23%. In addition, there was a significant positive correlation between perceived social support and both gender and having a physicians' medical license.

In fact, the level of social support varies across different regions in China, with more developed areas providing higher levels of social support than less developed areas. For example, standardized training physicians in Guangdong Province (60.3%) report greater satisfaction than those in Gansu Province (30.4%) do (4, 18). There is a lack of regionalized empirical data on social support for pediatric resident physicians undergoing standardized training in Southwest China. In our study, the percentage of residents satisfied with the overall social support they received during their residency training was 63.68%, which is consistent with the local economic level. Interestingly, in the U.S., 93.6% of residents reported high satisfaction with and social support in their training programs (19). This may account for the effect of economic level in this phenomenon (20).

In this study, women reported higher scores for utilization of support (*P* < 0.05), which suggests that female residents may be more likely to seek and benefit from social support during their training than male residents. This finding aligns with previous research indicating gender differences in coping strategies and emotional expression (21). Compared with their male counterparts, female participants reported slightly higher levels of objective social support, subjective social support and total support, although the difference was not statistically significant (*P* > 0.05). Further analysis revealed that gender was an independent variable significantly associated with social support. Female pediatric residents were three times more likely to be satisfied with the level of social support than male pediatric residents in this study (OR = 3.12; 95% CI: 1.75–5.23; *P* < 0.001). Previous studies have suggested that women, while balancing work, are also expected to undertake more family responsibilities than men are, making them more susceptible to external disturbances and prone to low mood, exhaustion, and tension. As a result, they experience more severe burnout and receive less social support (20, 22, 23). This survey, however, found that female pediatric resident physicians reported significantly higher levels of social support than their male counterparts. On the one hand, this reflects social progress and increased care for women; on the other hand, it highlights the neglect of male resident physicians' mental health and social support. This may also be related to the traditional Chinese cultural expectations of men, such as a greater reluctance to express emotions, higher familial expectations, greater economic pressure, and fewer outlets for emotional sharing (24). Therefore, social support for male pediatric resident physicians needs to be strengthened, and further research in this area is warranted.

Another interesting phenomenon revealed by the survey was that having obtained a physician's medical license was associated with higher levels of satisfaction with social support and thus was a factor influencing social support, consistent with the opinion of previous researchers (25, 26). Under national policy, vigorously promoting the “Four Certificates in One” initiative—which integrates graduation certificates, degree certificates, physicians' medical license, and standardized residency training certificates—passing a medical practitioner qualification examination has become a crucial component of the residency training process. Only those who successfully pass this examination are eligible to apply for the final residency training assessment and obtain a standardized residency training certificate (27). In addition, possessing a physician's medical license enhances a resident's professional identity and career efficacy, enabling them to become more embedded in both the formal organizational structure and informal peer support networks, thereby obtaining broader and deeper social support across institutional, organizational, and interpersonal levels (28).

Furthermore, our results demonstrated that pediatric residents employed by the Children's Hospital of Chongqing Medical University had higher scores for objective social support and satisfactory social support. These resident physicians are more likely to gain recognition from their colleagues, earn the trust of supervisors, and receive greater institutional support from the hospital, thereby broadening and deepening their social support network. However, there were no differences in social support for education, level of training or annual income among these pediatric residents. Interestingly, despite significant variations in income among different levels of medical trainees (medical students, residents, and early-career physicians), burnout remains highly prevalent and consistently severe across all these groups (29). This phenomenon may be attributed to improved modern family conditions, which have reduced the relative emphasis on income concerns, while training-related stressors appear to exert a more universal impact.

This study, while providing valuable insights, is subject to several limitations. First, all the patients in this study were recruited exclusively from the Children's Hospital of Chongqing Medical University, resulting in a relatively limited sample size. Future studies involving larger and more diverse multi-center cohorts could increase the statistical power and generalizability of the findings. Second, the use of online questionnaires has several limitations. Owing to the anonymous nature of the responses, we cannot verify whether the intended participants completed the survey or whether the link was shared with and responded to by individuals outside the target group. Finally, we did not collect data to explore the underlying reasons why certain pediatric residents reported inadequate levels of social support. By addressing these limitations, future studies can further refine our understanding of social support for pediatric residents, and the results can inform the development of more effective interventions to support these residents' mental health and professional development. Our study also found lower social support among male residents. Future research could use qualitative methods like interviews or focus groups to better understand the specific barriers these men encounter.

## Conclusions

To our knowledge, this is the first survey on the social support of pediatric residents in Southwest China. The findings contribute to the understanding of the social support status of pediatric residents in this region. The identification of gender differences in social support highlights the importance of tailored interventions to address the specific needs of male pediatric residents. Furthermore, the correlation between obtaining a physician's medical license and receiving increased social support underscores the value of national policies promoting comprehensive residency training. The recognition of institutional factors, such as employment at the Children's Hospital of Chongqing Medical University, that influence social support emphasizes the role of organizational culture and support networks in promoting the wellbeing of pediatric residents.

## Data Availability

The raw data supporting the conclusions of this article will be made available by the authors, without undue reservation.
